# National plans: case study Belgium

**DOI:** 10.1186/1750-1172-7-S2-A3

**Published:** 2012-11-22

**Authors:** Ri De Ridder

**Affiliations:** 1 IHDI National Institute for Health and Disability Insurance (RIZIV INAMI Belgium

## 

In October 2011 the Belgian Fund for Rare Diseases and Orphan Drugs, a consortium of stakeholders supported by the King Baudouin Foundation, handed over the recommendations and proposed measures for a Belgian Plan for Rare Diseases. In follow-up of the EU recommendations to issue national plans by 2013, the Minister of Public Health and Social Affairs commissioned the Fund to propose a comprehensive plan. The proposals cover 11 action domains, among them patient empowerment, improving access to treatment, ethics and governance. 42 measures have been proposed and a budgetary estimate was issued. Realising the plan would necessitate a 17 million euro investment over a 5 year period and an additional yearly expenditure of 44 million euro: compared to the national health Insurance budget of 25 billion euro, this seems a limited effort to realise! Still, adding expenditure to the budget under actual pressure to curb down health expenditure growth rates is not evident. Although governmental endorsement of a national plan has not yet taken place, most probably due to the economic and budgetary context, several measures have been launched by the Minister. These measures pertain to the core of action to be taken: setting up a national registry for rare diseases, disclosing information through Orphanet in national languages, developing centers of expertise based upon international guidelines, recommendations and guidance through Eucerd, adapting procedures in order to make early temporary reimbursement possible.

